# Supplementary material for: An atlas of human kinase regulation
Source: Mol Syst Biol. 2016 Dec 1;12(12):888. doi: 10.15252/msb.20167295 (PMC5199121; doi:10.15252/msb.20167295)
Supplement: Supplementary file 1 — Appendix [file MSB-12-888-s001.pdf]

Appendix: An Atlas of Human Kinase Regulation

David Ochoa[1], Mindaugas Jonikas[2], Robert T. Lawrence[3], Bachir El Debs[4], Joel Selkrig[4], Athanasios Typas[4], Judit Villén[3], Silvia Santos[2], Pedro Beltrao[1*]

[1] *European Molecular Biology Laboratory, European Bioinformatics Institute (EMBL-EBI), Hinxton, United Kingdom*

[2] *Quantitative Cell Biology group, MRC Clinical Sciences Centre, Imperial College, London, United Kingdom*

[3] *Department of Genome Sciences, University of Washington, Seattle, USA*

[4] *European Molecular Biology Laboratory (EMBL), Genome Biology Unit, Meyerhofstraße 1, 69117 Heidelberg, Germany*

[*] Correspondance to Pedro Beltrao: pbeltrao@ebi.ac.uk

Table of contents

- Appendix Figure S1
- Appendix Figure S2
- Appendix Figure S3
- Appendix Figure S4

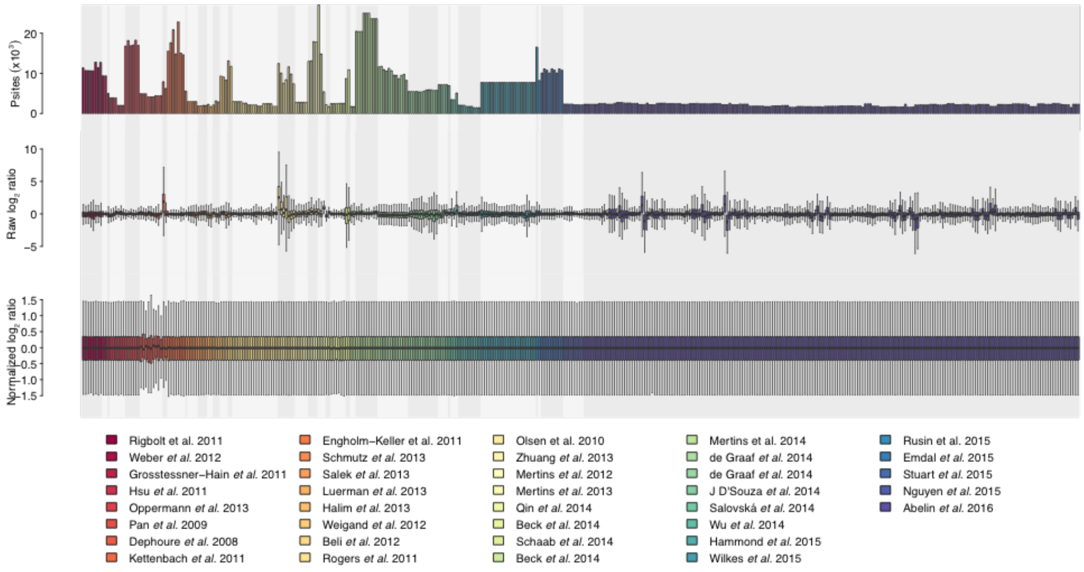

Appendix Figure S1 - Conditional quantitative phosphoproteome normalization.
Total number of modified phosphosites - top- , distribution of phospho-proteomic profiles before - middle - and after - bottom - quantile normalization. Conditional perturbations are grouped by publication and ordered chronologically. For clarity, only articles passing quality control criteria are shown.

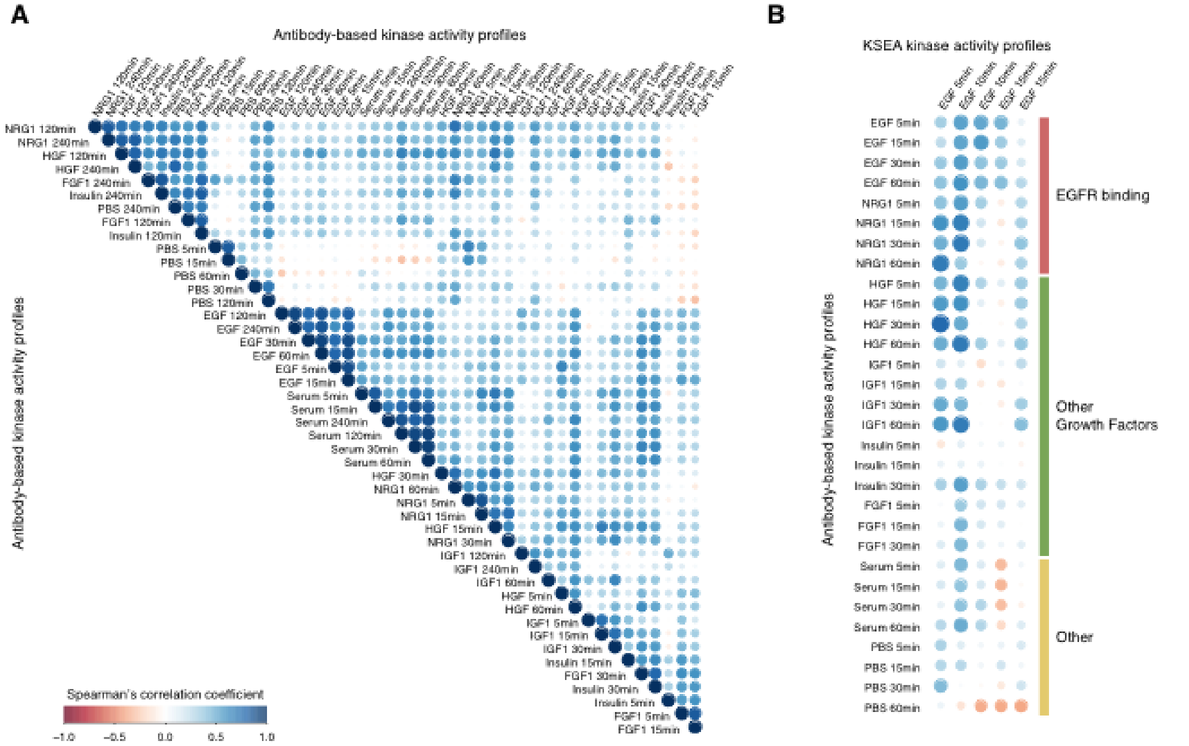

Appendix Figure S2 - Agreement between RPPA and KSEA kinase activities after perturbation of BT20 cells with different ligands.

A Pearson correlation between the profiles of 48 phospho-protein quantifications at different time points after stimulating BT20 cells with different ligands. The phosphosites were quantified using Reverse Phase Protein Array (RPPA) and normalized to make the quantifications comparable across conditions and antibodies. RPPA data was part of the HPN-DREAM breast cancer network inference challenge.

B Pearson correlation between the kinase activity response of 18 kinases, when inferred using KSEA from shotgun proteomics or quantified using RPPA early after EGF stimulation. Ligands are split depending if they stimulate directly the EGF receptor, they can also act as growth factors and potentially have similar downstream consequences or neither of those.

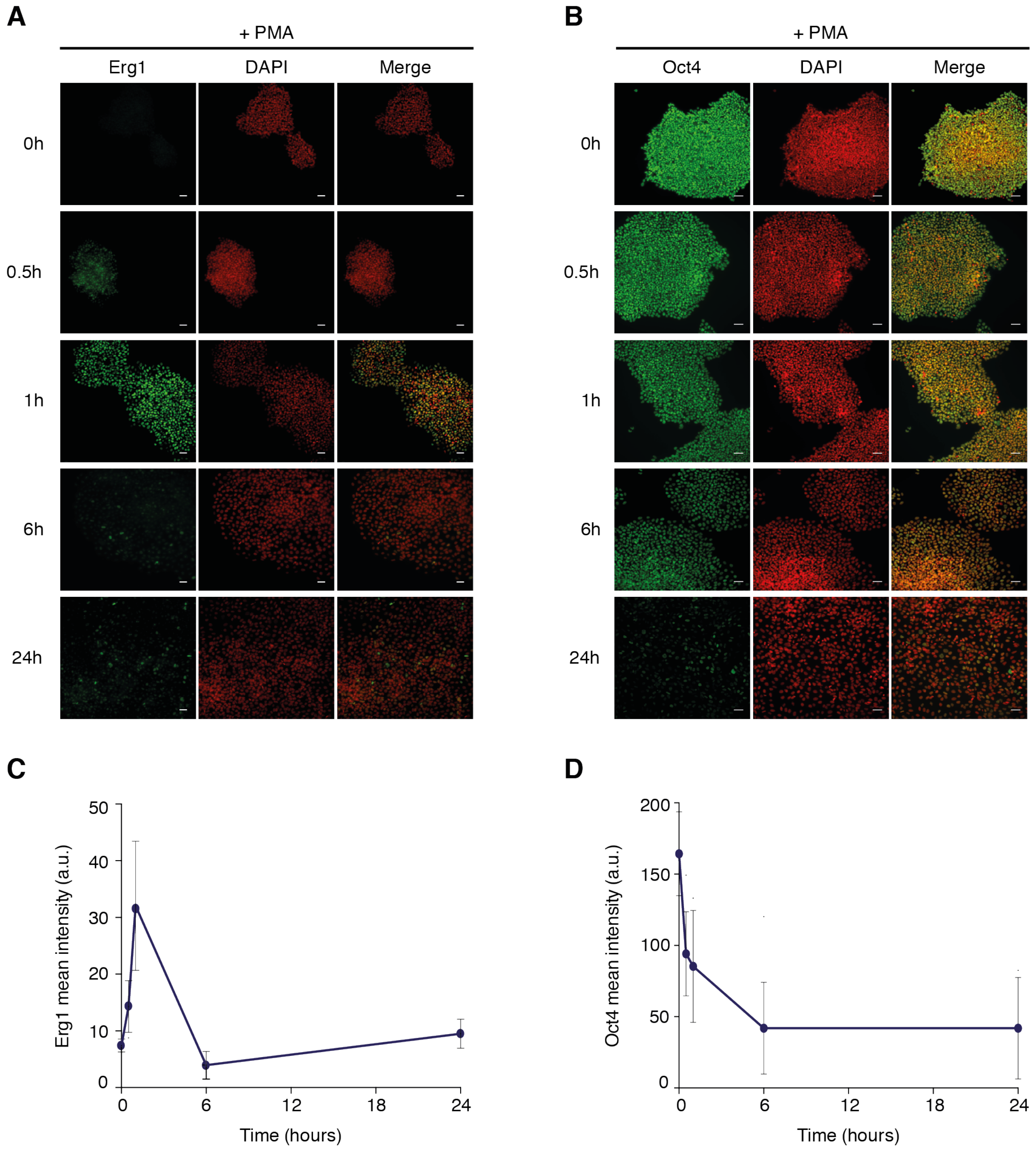

Appendix Figure S3 - Erg1 and Oct4 as early and late markers of PMA-driven differentiation of hES cells.
A-B Representative images of Erg1 and Oct4 - respectively - expression in hES cells stimulated with PMA. Scale bar: 30μm.
C-D Time course quantification of Erg1 and Oct4 - respectively - expression levels after PMA stimulation. Error bars represent mean ± SD. n=2500 cells per experimental time point.

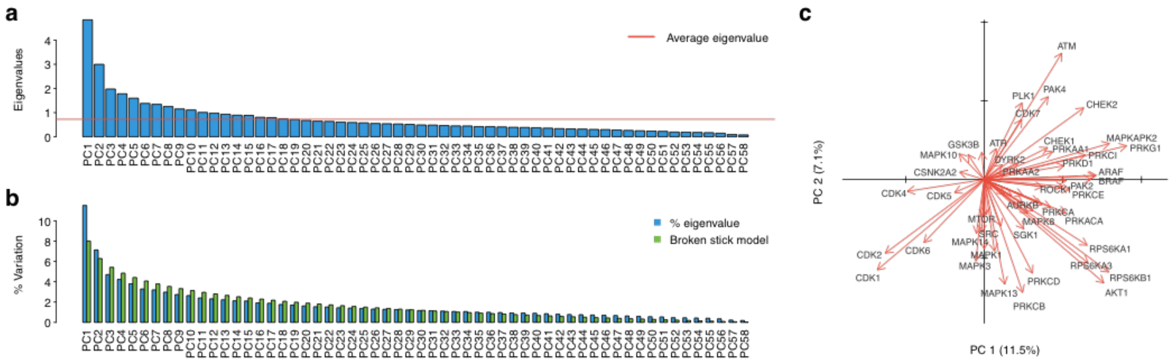

Appendix Figure S4 - PCA-based map of kinase signaling states.

A Eigenvalues for each of the 58 components relevant to define the number of significant components based on the Kaiser criterion. Red line displays average eigenvalue.

B Percentage of variation explained in each of the 58 components compared with these obtained applying broken stick model.

C Factor loadings corresponding to each of the 58 kinases displayed in the first 2 components. For clarity, some kinase labels are not shown.
