# Supplementary material for: An atlas of human kinase regulation
Source: Mol Syst Biol. 2016 Dec 1;12(12):888. doi: 10.15252/msb.20167295 (PMC5199121; doi:10.15252/msb.20167295)
Supplement: Supplementary file 2 — Expanded View Figures PDF [file MSB-12-888-s002.pdf]

Expanded View Figures

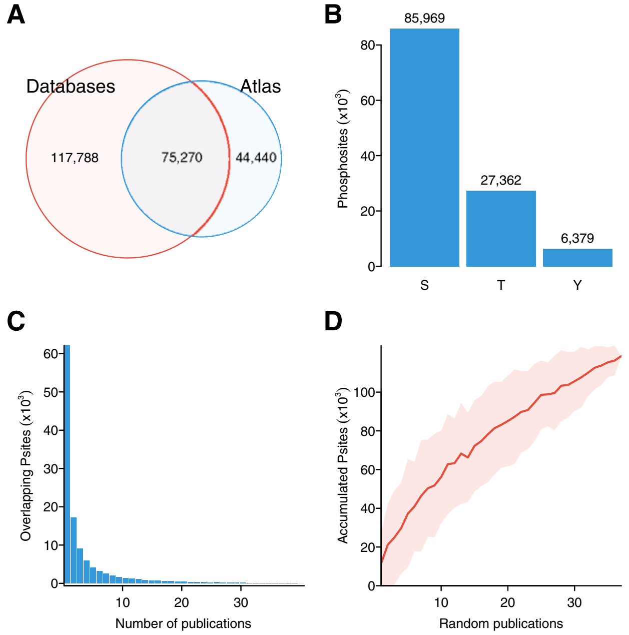

Figure EV1. A map of human conditional phospho-regulation.

- A Venn diagram including the total number of phosphorylated residues for which quantifications were collected and the number of sites contained in the curated phosphosite databases: PhosphoSitePlus, HPRD, and Phospho.ELM (August 2014).
- B Total number of quantified serines (S), threonines (T), and tyrosines (Y).
- C Distribution of sites reported by multiple parallel publications.
- D Accumulation of sites as publications are randomly aggregated (100 permutations). Red line shows mean and the shadowed area mean $\pm$ 1 standard deviation.

Figure EV2. Agreement between immunofluorescence and KSEA activities during hESC differentiation.
For each of the 10 kinases under study, the panel of phospho-specific antibodies report the activity at 0, 30, and 60 min after PMA stimulation (left panels). Data represent mean $\pm$ SD. A minimum of 1,000 cells were analyzed in each experimental condition. The KSEA activities are inferred from the MS-quantified substrates in the time intervals 0–30 and 0–60 min after PMA stimulation and represented as a fraction of the theoretical limit based on the number of KSEA permutations. Note that the MS-based horizontal bars represent a time interval (i.e. 0–30 min) as compared to the IHC data measured in each individual time point (i.e. 30 min). For both time intervals, the relative position of each of the known substrates within the quantitative phosphoproteomic profile indicates enrichments on up-regulated (blue) or down-regulated (red) phosphosites (right panels).

© 2016 The Authors

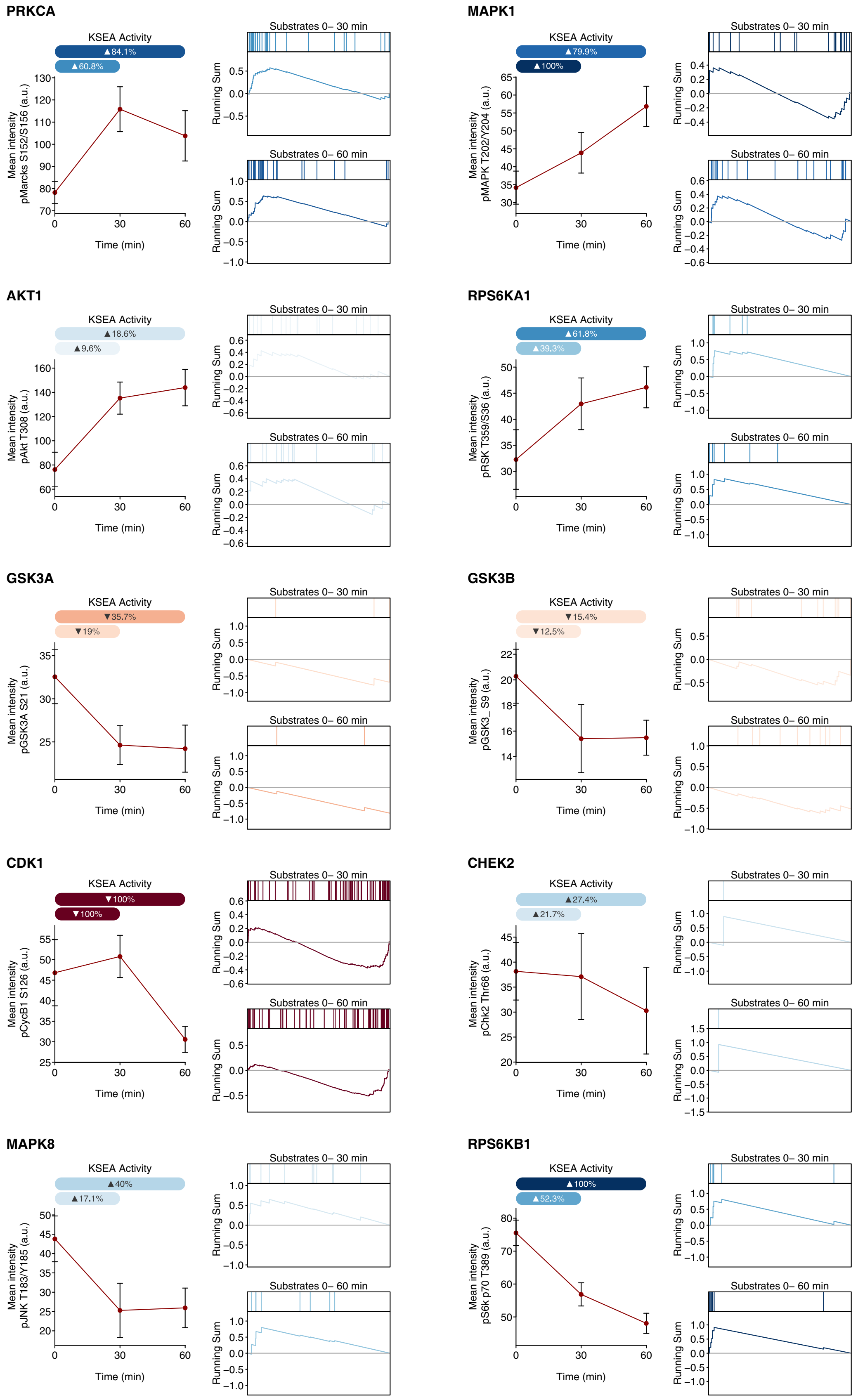

Figure EV2.

 © 2016 The Authors

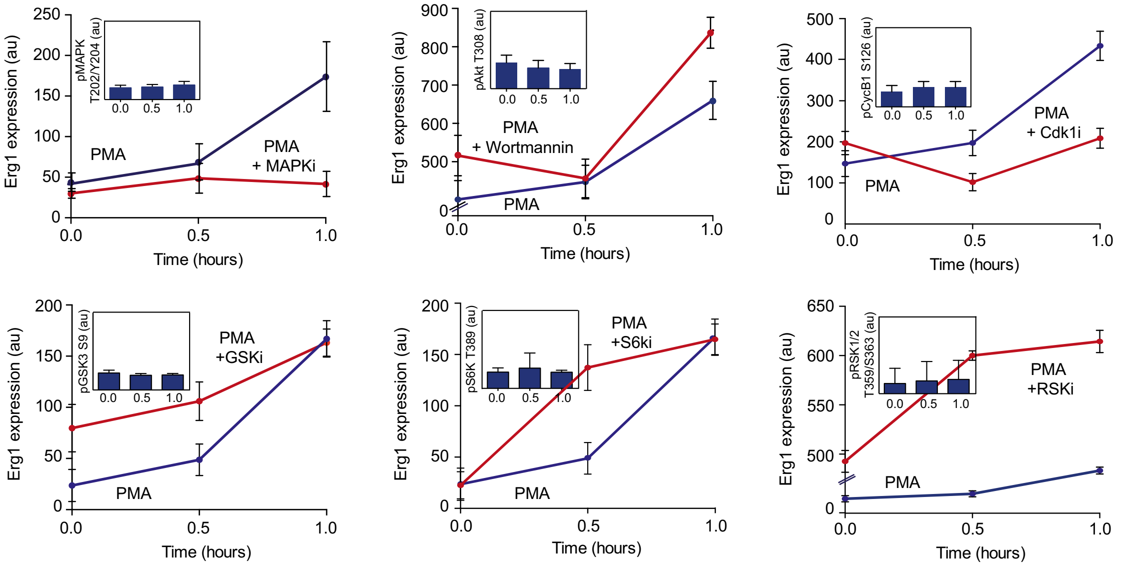

Figure EV3. Time course quantification of Erg1 expression levels after PMA stimulation in the presence of kinase inhibitors.
Inset displays kinase activity measured with phospho-specific antibody after inhibitory treatment. Data represent mean ± SD (n > 1,000).

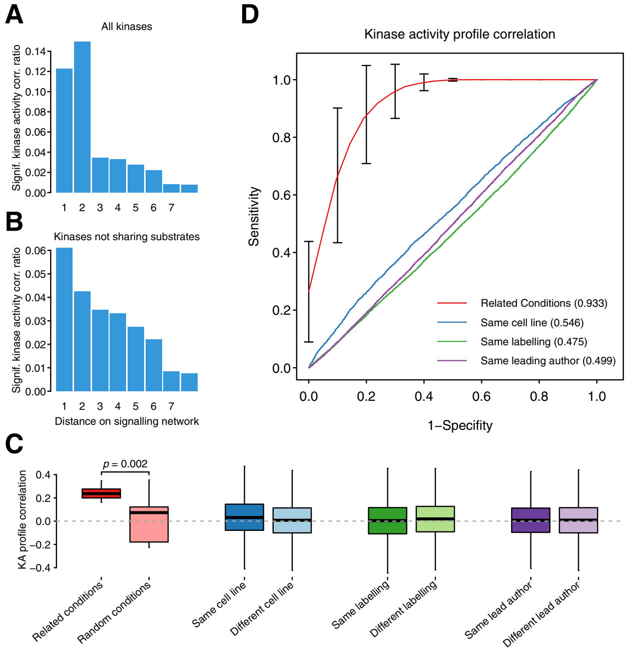

Figure EV4. Kinase activity profiles as signatures of the molecular response.

A Fraction of significant correlations (FDR < 0.05) when comparing the activities across conditions for kinases at different distances in the signaling network. The network contains all kinase–substrate interactions from PhosphoSitePlus, HPRD, and Phospho.ELM.

B Same as (A) but excluding kinase pairs sharing substrate residues.

C Differences in correlations of kinase activity (KA) profiles grouped by biological or technical origin. The activity profiles for the 215 kinases are correlated by sample. The correlations between pairs of conditions assayed in different studies are grouped based on biological or technical criteria. Related conditions published in independent studies present significantly different correlations (Student's t-test, P = 0.002), than the same number of random pairs of conditions also published in different studies. The boxes represent the 1st, 2nd (median) and 3rd quartiles and the whiskers indicate 1.5 times the IQR.

D ROC curves denoting the predictive power of the same kinase activity profile correlations to discriminate between related conditions in different studies from random pairs of conditions (mean AUC = 0.933), Error bars display SD; n = 100 iterations, pairs of conditions assayed using the same cell line against different cell line (AUC = 0.546), same labeling methods versus different technique (AUC = 0.475), or samples coming from different publications with the same leading author against different leading authors (AUC = 0.499).

© 2016 The Authors

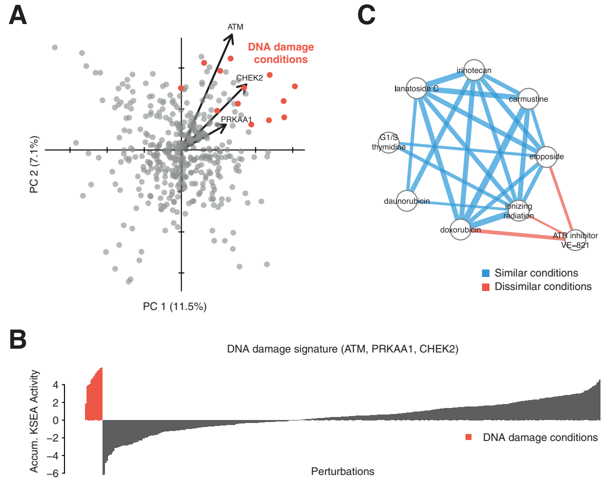

Figure EV5. Kinase activity profiles identify DNA damage responses.

A All conditions displayed on the PCA first two components. Perturbations potentially producing DNA damage are highlighted in red. Vectors report the loadings of three relevant kinases.

B Accumulated KSEA activity of the DNA damage biomarkers ATM, PRKAA1, CHEK2 across 399 perturbations.

C Network displays significantly correlated conditions. Blue edges report significant correlation between conditions involving damaging conditions. Red edges report significant anti-correlations with ATR inhibition, a kinase involved in the DNA damage response. Edge width represents the strength of the correlation.

 © 2016 The Authors
